# Autoethnographic Insights from Neurodivergent GAI “Power Users”

**DOI:** 10.1145/3706598.3713670

**Published:** 2025

**Authors:** Kate Glazko, JunHyeok Cha, Aaleyah Lewis, Ben Kosa, Brianna L Wimer, Andrew Zheng, Yiwei Zheng, Jennifer Mankoff

**Affiliations:** University of Washington, Seattle, Washington, USA; Department of Statistics, University of Wisconsin – Madison, Madison, Wisconsin, USA; Paul G. Allen School of Computer, Science and Engineering, University of Washington, Seattle, Washington, USA; Department of Computer Sciences, University of Wisconsin – Madison, Madison, Wisconsin, USA; Department of Computer Science and Engineering, University of Notre Dame, South Bend, Indiana, USA; Independent Researcher, Fremont, California, USA; National Coalition of Independent Scholars, Battleboro, Vermont, USA; Allen School of Computer Science and Engineering, University of Washington, Seattle, Washington, USA

**Keywords:** auto-ethnography, generative artificial intelligence, accessibility, neurodivergent people, intersectionality, stigma, Human-centered computing → Accessibility technologies, Natural language interfaces

## Abstract

Generative AI (AI) has become ubiquitous in both daily and professional life, with emerging research demonstrating its potential as a tool for accessibility. Neurodivergent people, often left out by existing accessibility technologies, develop their own ways of navigating normative expectations. GAI offers new opportunities for access, but it is important to understand how neurodivergent “power users”—successful early adopters—engage with it and the challenges they face. Further, we must understand how marginalization and intersectional identities influence their interactions with GAI. Our autoethnography, enhanced by privacy-preserving GAI-based diaries and interviews, reveals the intricacies of using GAI to navigate normative environments and expectations. Our findings demonstrate how GAI can both support and complicate tasks like code-switching, emotional regulation, and accessing information. We show that GAI can help neurodivergent users to reclaim their agency in systems that diminish their autonomy and self-determination. However, challenges such as balancing authentic self-expression with societal conformity, alongside other risks, create barriers to realizing GAI’s full potential for accessibility.

## 1 Introduction

The use of Generative AI (GAI) has become widespread in many facets of life. In the workplace, it is used for tasks such as software development [8, 24, 28, 87, 113], while businesses are integrating GAI into areas like customer service [15] and hiring [16, 44, 118]. GAI is also being explored as a tool for creative tasks, including design and artwork [19, 140]. One burgeoning area of impact for GAI is accessibility. The BeMyEyes app, which recently integrated a GAI image description component, is already used by more than 750,000 blind and low vision (BLV) people [9]. Additionally, a number of disability- or accessibility-focused GAI tools have been deployed on the popular OpenAI GPTs Store [109]. Many of these GPTs have active engagement and high rankings. ADHD Companion, a self-help GPT for people with ADHD, had over ten thousand conversations and a 4.5 rating at the time this paper was written; Autism Support had over five thousand conversations and a 4.7 rating; Therapist/Psychologist-Fictional had over one million conversations. Both the deployment and engagement with these tools demonstrate that GAI for accessibility is not a promise or vision but a current reality. Disabled people are engaging with these commercially available technologies despite documented shortcomings (i.e. disinformation [11], built-in biases [44, 58, 154], and lack of accessible validation methods [1, 24, 44]), to create more navigable and accommodating spaces in their daily lives [1, 45].

However, we know very little about GAI’s use by disabled people over time. Research has begun to document how neurodivergent people use GAI to meet access needs, primarily through analyses of online discussions, identifying common use cases such as emotional regulation, communication support, and productivity aids (*e.g.,* [20]). Emerging work suggests that only a small minority of students use GAI daily [62]. Students with disabilities appear more likely to report daily use [41], yet the majority of participants in studies around GAI use for accessibility are not characterized as frequent, sustained, or expert users of GAI (*e.g.,* [25, 41, 42, 45, 65, 138]). Studying the long-term, “power use” of GAI is important for understanding how these emergent technologies are integrated into accessibility practices over time. Examining sustained use would reveal not just how users mitigate limitations and harms [1, 20, 24, 139], but whether GAI helps users navigate systemic barriers in their daily lives or ultimately reinforces ableist expectations. Popular media has featured longitudinal GAI use, but necessarily among people comfortable with publicly disclosing their use of GAI and their disability identities [54]. There remains a significant lack of understanding around the long-term experiences of those who rely heavily on GAI for accessibility but choose to keep their disability or GAI use private.

This work seeks to bridge this gap by capturing perspectives from neurodivergent individuals with stigmatized disability identities who are not just casual users but “power users” of GAI. Through collaborative autoethnography, supported by a GAI-assisted anonymous diary and interview component, we capture the ways in which “power users” have been integrating GAI into their lives and work to meet accessibility needs. Our autoethnography team (which includes all authors of this paper) represents experiences of people who identify as neurodivergent, a disability domain whose access needs have largely not been addressed by existing accessibility technologies or social solutions [12, 89]. In addition, our team of authors includes multiply-disabled individuals with varied racial, cultural, and language backgrounds. Our research aims to allow findings to emerge inductively from the collected data, reflecting the lived experiences and varied needs of a diverse group of neurodivergent GAI users.

Through our reflections, we aim to provide a deeper insight into GAI’s real-world impact on accessibility for neurodivergent, expert users. Our work demonstrates the positive impacts on access engendered by the agency and control provided by GAI over which accessibility needs are met and how they are met. We found uses of GAI for saving time and cognitive energy, communicating and conforming, substituting for people to reduce social costs, and understanding and consolidating information.

By analyzing the data not only through the lens of personal usefulness of accessibility technologies but also through the frameworks of self-determination [29], stigma, camouflage, masking, and intersectional experiences of neurodivergence, we offer new insights into the limitations and trade-offs of GAI use. For example, the authors were self-aware in choosing GAI despite its risks (i.e. errors, bad advice [139], dependence [24, 63], potential negative impacts on learning [24], and built-in biases [24, 44]), carefully negotiating these risks in response to implicit societal pressures to conform to hegemonic ideals of timeliness, productivity, behavior, and language. While technology aids in shaping access, true accessibility extends beyond the individual and requires addressing ableist and racist systems. Ongoing epistemic injustices against neurodivergent people persist [39, 72, 96, 159] and cannot be resolved through GAI use. Our work explores the strengths and limitations of GAI as an access tool, offering critical insights into the broader societal pressures shaping access needs addressed by GAI, trade-off of use, concerns, and hopes for the future.

## 2 Background

Since becoming widely available to the public, GAI has steadily gained recognition for its potential as a tool for accessibility. Glazko et al. conducted the first autoethnography on GAI and its risks and benefits to accessibility with a diverse group of seven authors, five of whom identified as disabled [45]. Creative image generation, information extraction as a tool to support brain fog, communication support for autistic users, and GUI description have been described as promising use cases for accessibility [45]. Since then, studies have investigated how specific populations use or could use GAI, including autistic people [20, 25, 65], blind people [1, 158], and people with intellectual and developmental disability [51]. For example, studies have found that blind and visually impaired individuals use GAI to ‘offload’ cognitively demanding tasks [158], obtain personal help such as fashion advice [158], and create content or retrieve information [1]. Additionally, research has explored the use of GAI for designing accessibility tools, such as co-designing accessible instruments with disabled musicians [5].

Notably, and unlike most prior accessibility research [89], many recent papers about disabled GAI use emphasize disabilities that are often grouped under the label “neurodivergence.” Neurodivergence refers to those whose cognitive ability profiles or neurology diverge from the dominant societal standards of “normal” [83, 148] and can include autism, ADHD, dyslexia and other learning disabilities, neurodevelopmental differences, and some mental health conditions. Neurodivergent people often face epistemic injustices [83], such as social exclusion or lack of access to knowledge, in a self-reinforcing cycle [39, 83, 96]. Given these injustices, where lived experience, knowledge, and neurodivergent people’s needs are devalued in favor of conformity to normative expectations [50]– it is no surprise that social stigma [46] and camouflaging [106, 111] to meet such expectations are both common in the lived experience of neurodivergence, as described in more detail in Section 2.2. Although interactions with chatbots have been used as a source of stigma-free support in even early versions of AI chatbots [58, 95], there remains limited knowledge of how GAI use impacts the ways in which neurodivergent people navigate stigma, normative expectations, and accessibility barriers over time.

In the remainder of this section, we summarize what is known about GAI use and neurodivergence, as well as neurodivergent use of accessibility technology more generally; discuss the role of stigma camouflaging and masking in neurodivergence, and summarize what is known about intersections of neurodivergence and other identities, a commonly overlooked concern in accessibility research [52].

### 2.1 Accessibility technology for neurodivergence

Existing research on accessibility technology (AT) designed for neurodivergent adults has driven critical reflections on how and whether the development of those technologies is motivated by lived experience and expressed needs– or whether it simply attempts to force neurodivergent people to conform to allistic norms [132, 152]. For example, Spiel et al. describe how most AT for ADHD is geared towards diagnostics or disciplining users to behave in more neurotypical ways [132]. They describe a lack of AT designed for adults with ADHD and detail how future AT could help them meet the demands of a neurotypical society– challenges ADHD adults already address through non-AT coping strategies [132]. Williams and Gilbert’s survey of wearable technologies for autism found that only 10% of them met autistic people’s expressed needs, such as helping with sensory/emotion regulation, communication, or executive function, while the other 90% centered on shaping behavior to appear more neurotypical [152].

As a result, recent works have begun to explore how neurodivergent adults creatively and methodically construct their own access solutions. Williams and Park highlight self-agency in autistic people’s design of their own supports in areas such as executive functioning and emotional regulation, by leveraging technologies such as digital reminders and calendars, in combination with peer support networks and environmental modifications [153]. This draws on Self-Determination Theory, which frames autonomy, competence, and relatedness as essential drivers of wellbeing [29], emphasizing that accessibility tools should prioritize user empowerment by enabling self-directed control rather than enforcing conformity to normative expectations [150]. Similarly, work has documented digital apps and tools used by ADHD students for meeting the demands of their schoolwork and managing life tasks such as organization and financial planning [35]. Indeed, studies of community-created solutions highlight innovative answers to access barriers that are overlooked by mainstream literature, such as the use of “body doubling” to generate momentum and stay on task by sharing presence, or digital analogs of presence, with one another [31, 32]. Online communities (i.e., Instagram, Reddit) provide opportunities for neurodivergent adults to find validation and acceptance from peers, explore treatment options, and navigate social tensions or work interactions [30, 40, 68, 147]. Some neurodivergents have embraced interactions with chatbots as a method of meeting social or emotional needs or as a way of escaping stigma [30, 63, 77, 95], which will be explored more in the next section.

Studies of neurodivergent AI use have similar themes. For example, one study explored how autistic people would like to use GAI to navigate daily life and social experiences [25], communication in professional settings [65], or their attitude towards using deepfake technologies for simulating normative behaviors [42]. Similarly, Çarik et al. analyzed discussions from neurodivergent communities on Reddit, identifying common uses of LLMs for emotional support, communication assistance, and workplace productivity [20], while highlighting how neurodivergent individuals experiment with GAI prompting and share workarounds to address the neurotypical biases embedded in AI-generated responses. Other research moves beyond observational studies to evaluate specific GAI use cases: supporting communication of autistic workers [65], enhancing reading comprehension for people with ADHD [138], and generating images for people with intellectual and developmental disabilities [51]. However, these works primarily focus on specific incidents of use or structured tasks, leaving a gap in understanding how neurodivergent people confident with the technology integrate GAI into their strategies for navigating inaccessible systems and environments over time.

### 2.2 Bias, stigma, camouflaging and masking

Neurodivergent people often face social rejection [38, 67, 128, 129, 141], and social stigma [12, 74, 81, 102]. Autistic people, for example, are more likely to face discrimination in hiring and work [66], or biases from peers in academic settings [99, 137]. To overcome bias and other forms of stigma, neurodivergent people may seek to camouflage, or mask, non-normative traits associated with their condition [111]. Masking, or concealing a disability, can help in navigating an ableist world [46]. Masking can also be a strategy to avoid adverse social experiences and achieve success and acceptance [2, 106], described as a necessity to survive in normative conditions [13]. Yet masking is accompanied by its own set of risks such as exhaustion, loss of identity, [13], and increased suicidality [23]. Closely tied to masking is disclosure. While disclosure of disability should ideally lead to improved support, accessibility, and other positive outcomes, instead, disclosure of disability to peers and colleagues can result in further discrimination [99]. Many neurodivergent people have the option to hide their disability, and as a result of these risks, choose to disclose primarily in safe online communities [40, 147] or in interactions with chatbots [63, 77, 95].

The arrival of GAI-based chatbots could further improve the value of this support mechanism. Factors such as self-stigma can make non-human sources of support such as AI chatbots more desirable than human support [58]. Chatbot use has demonstrated benefits such as reduced loneliness and decreased suicidality [95]. However, the use of chatbots also poses risks such as over-dependence [25, 63] or chatbots giving harmful advice [139].

Additionally, stigma and bias are concerns in interactions with GAI. GAI exhibits ableist bias when asked to complete basic writing prompts about individuals with disabilities [53, 64]; classifies disability-containing phrases as toxic [64]; perpetuates harmful stereotypes [37, 45]; and portrays disability as lonely or even horrific [92]. These built-in biases are particularly prevalent and severe surrounding neurodivergence [14, 44]. A resume audit demonstrated bias towards resumes containing disability-signaling items such as awards and scholarships, with the bias notably worse towards neurodivergence terms such as autism and depression [44]. Neurodivergence-related terms were negatively associated with words such as honesty and were positively associated with danger, badness, and other negative concepts in multiple AI language models in one study of language models [14]. Given these potential risks and built-in biases, it is essential to understand how neurodivergent people navigate trade-offs of GAI use against their access and support needs, particularly when using GAI to mitigate stigma and social rejection.

### 2.3 Intersectional experiences of neurodivergence

The experience of being neurodivergent can be inextricably impacted by other intersectional identities such as race and ethnicity [4, 43, 82, 84, 119, 130], gender [7, 48, 49, 69, 78, 82, 130, 156], and LGBTQIA+ status [57, 100, 130]. Racial identity [10, 26, 34] and gender identity [115] can impact timely diagnosis of conditions like ADHD and autism, impacting adequacy of treatment [26] and subsequent life outcomes and wellbeing [36, 73, 107, 146]. Racial biases, for example, contribute to underdiagnosis of neurodivergence [34, 94] and over-punishment of non-normative behaviors in people of color, leading to the “learning disability to prison pipeline” [94]. Le, in her auto-ethnography of navigating disability and racial identity, describes how language and cultural barriers impacted her family’s ability to access neuropsychology services [34]. Similarly, gendered stereotypes around the presentation of ADHD and autism impact the diagnosis of women and result in increased masking and anxiety [7, 48]. Conversely, an autism diagnosis can impact access to gender-affirming care services due to ableist assumptions [122].

Beyond diagnosis and treatment, neurodivergent people who are non-white can face extra challenges navigating typically-white normative standards of language and behavior [43, 82, 110, 120, 134]. One strategy employed by people minoritized within their social or environmental contexts is code-switching [6, 157]. Code-switching refers to the linguistic and behavioral adaptations individuals make to their speech, appearance, and expression to fit prevailing norms [104, 105, 135], to reduce exclusion and navigate social power dynamics [22, 98]. Code-switching is well known in Black communities/African American Vernacular English speakers (AAVE) [56], but occurs across various ethnic and racial groups. Code-switching can negatively impact mental health and is exhausting [55, 125, 155]. These negative impacts are further amplified when paired with other conforming behaviors– for example, Black neurodivergents in academia face increased exhaustion and self-suppression due to the combination of code-switching and masking [84], leading to risks such as trauma and further marginalization [119]. Lewis and Arday, through auto-ethnography, describe the challenges of not only having to mask in academia– but to do so in the face of inequity of treatment compared to white, neurodivergent academics [84]. Despite its harms, code-switching–like masking–remains an important strategy for navigating environments where one experiences marginalization and exclusion [135].

Intersecting identities, such as those illustrated above, can increase the stigma and marginalization that neurodivergent people experience, limiting their ability to seek support. Le describes how immigrant and generational status of her Vietnamese parents resulted in shame around discussions of mental health and neurodivergence, as well as the increased isolation experienced by her mother [82]. Someki et al. detail how autistic Japanese college students face more stigma from their peers than autistic U.S. college students due to cultural norms around collectivism [130]. Culture can even impact the acceptance of accessibility technology (AT) use. Li et al. highlight negative Chinese cultural attitudes around AT result in reduced or hidden use of AT to “save face”, or preserve personal self-esteem by signaling minimal help from AT [85].

The increased challenges that neurodivergent people with intersectional identities face (e.g. increased camouflaging behaviors including masking, code-switching, and navigating cultural and familial norms) can in turn lead to negative impacts [23, 69, 82, 84, 123]. Despite these risks, conforming to normative communication standards through behaviors like masking and code-switching remains an often-coerced necessity [127] to reduce further exclusion from institutions such as academia [84, 135] or the workplace [116], and reduce social stigma [106, 127, 135]. Yet, a significant gap remains unaddressed—most HCI research fails to consider the intersection of multiple identities [126]. Accessibility research often overlooks the influence of racial identity or ethnicity on the lived experience of disability and, consequently, the interaction with AT [52]. Existing research on GAI use by neurodivergent people [25, 45, 65] does not deeply explore additional social pressures faced by people with intersectional identities, and whether GAI plays a role in navigating strategies such as code-switching or meeting dominant culture communication norms. Additionally, the impact of risks such as built-in racial [24, 60, 154] or linguistic biases [60] and lack of representation in GAI [24] on these strategies remains unknown.

## 3 Autoethnography Design and Rationale

In this autoethnography, we aim to highlight the benefits and trade-offs of skilled, long-term GAI use for neurodivergent people with diverse identities, which shape their experiences of stigma and the pressures to conform to societal norms and expectations. As a team of neurodivergent people who are successful college graduates with high-tech computer science-related positions, graduate students, or professors who also have a broad range of intersecting identities, we build upon the body of work of neurodivergent-focused research led by neurodivergent researchers [131, 132, 150, 153]. This is a group that tends to be underrepresented in computer science [17, 133]. This focus means that the careers and education of the team represent a very small segment of the broader neurodivergent community. However, it allows us to highlight the benefits and trade-offs of skilled, long-term GAI use and how it shapes the experiences of stigma and the pressures to conform to societal norms and expectations.

Prior work in disability studies and accessibility has used collaborative autoethnography to uncover nuanced and poignant insights into lived disability experience [45, 59, 90, 93]. Collaborative autoethnography addresses ethical shortcomings in single-author ethnographies, such as allowing those with less institutional privilege to contribute diverse perspectives with less fear of repercussions due to providing a greater degree of anonymity than single-author autoethnographies [79]. Additionally, it provides authors with agency over how their story is told through co-construction of knowledge, allowing authors to share their individual experiences and collectively illuminate common themes and divergent viewpoints within the group. We describe our method, team, and approach to supporting author privacy in more detail below.

### 3.1 Autoethnographic Team

Eligibility criteria for the autoethnography included identifying as disabled, neurodivergent, or having a mental/physical health condition and at least three months of consistent GAI use in daily life. Most authors surpassed this minimum criterion and had been actively using GAI well beyond the common, one-year window of assistive technology abandonment [112]. The earliest documented *first-time* use of GAI by an author was prior to December 2022, and the latest was in Spring of 2023.

Most of the authors initially tried out Generative AI (GAI) due to excitement, “*I like trying out new technologies and it was an up-and-coming piece of technology that I was really curious to try out*” (A5), or social influence: “*I saw a TikTok about it, I don’t remember what it was about but when I saw what the AI was capable of–answering any question in a humanlike way, I was super intrigued and instantly went to try it*” (A7). Several engaged with it to accomplish a specific task in their lives, “*the idea of making art just through text sounded great*” (A3). The frequency of GAI use varied across authors: interactions varied from less than a half hour of daily use, to as much as five hours of daily use (see Table 1). The team described the use of GPT, Gemini, Github Copilot, and unspecified GAI, with GPT being frequently mentioned in reflections (see Table 3). The team was established through a combination of serendipitous discussion, mutual connections, and snowball sampling.

Our final team includes eight U.S.-based authors, all with a college education in Computer Science with varying levels of experience in academia/industry. As such, our autoethnography emphasizes early adoption by a very specific subset of the neurodivergent community. Everyone in the team identifies as neurodivergent (N=8), and more than half identify with multiple conditions or disabilities (N=5), including having a mental health condition (N=4), having a disability (N=3) or having a physical health condition (N=2). Specific identities referenced by authors include ADHD, autism, obsessive-compulsive disorder, bipolar disorder, anxiety, social anxiety, fatigue, migraines, seizures, neurological disorders, color vision deficiency, chronic illness, cognitive impairment, and mobility-related disabilities and injuries. Authors represent a range of other identities, including different genders, racial identities, cultural identities, LGBTQIA+ status, and immigration status (see Table 2).

### 3.2 Data Collection Method

Starting in March 2024 and spanning through July 2024, each author participated in a one-month diary study [21] to capture reflections on their GAI use. Authors were instructed to write diary entries about any use of any GAI tools to meet access needs. At the end of the diary study, each author participated in an interview centered on our longitudinal use of GAI and our perceptions of its impact on our work and lives as neurodivergent people .

For the diary portion of the study, we instructed authors to journal their experiences through a custom ChatGPT chatbot, *DiaryGPT*^1^, created using the custom GPT graphical user interface [108]. *DiaryGPT*, available in the same interface as ChatGPT, provided a built-in [21], asynchronous way to report data– an important access need for our neurodivergent team [91]. *DiaryGPT* conversations were shared with the first authors through a google form. Participants were asked to share the privacy-preserving link already built into ChatGPT, optionally augmented with open-ended text; up to one link to relevant context, such as the original ChatGPT conversation that triggered the diary entry; and screenshots. When invoked, *DiaryGPT* asked a series of scripted questions about accessibility use of GAI (Appendix A). We created a similar chatbot, *InterviewGPT*^2^, for the interview at the end of the study. The two first authors piloted the interview prior to sharing it with the team. The interview focused on logistics of GAI use (length of use, quantity of use, tasks used for); satisfaction with GAI use; GAI’s ability to meet accessibility needs; and social interactions with GAI (Appendix B). The two first authors adapted the interview questions from an established methodology for exploring the sustained use of novel technologies [103]. In addition to diary and interview data, emails and chats about the study were used as data reflecting on the method, with explicit permission.

The use of GAI for elicitation diary study data has been documented in prior work [86]. The use of GAI for collecting structured interview data builds on the studied ability of GAI to generate relevant, domain-specific questions, as shown in studies of product requirements interviews [47], medical school preparation [27], and motivational interviewing for smoking cessation [75]. *DiaryGPT* and *InterviewGPT* had the advantage of collecting data anonymously. Anonymization of data collection even among ourselves allowed authors to share unfiltered reflections around uses of GAI for sensitive topics such as mental health, communication, legal matters, and disability identity. However, sometimes the data still contained identifying details (i.e. diagnoses, career details). When requested, responses were further anonymized by the first authors prior to analysis by any additional authors.

Because of the relative newness of this methodological approach and in line with recommendations for flexibility in participatory design with neurodivergent people [97], we allowed for iteration on our DiaryGPT design early in the study. Additionally, in adhering to confessional ethnographic methods [121], we allow for self-reflection on and analysis of these iterations. The first iteration of DiaryGPT was instructed to abide by best practices in running diary studies following Carter et al. [21]. After several days of use, we received negative feedback about *DiaryGPT*’s tone: “*It’s like talking to a horribly bad therapist who doesn’t understand me at all and is saccharine sweet trying to get it at the same time.*” (A6) We replaced the existing prompt with the prompt and questions in Appendix A. Most authors switched and expressed satisfaction with the updates “*New diary seems better. Less therapy session like for sure, more researcher like*” (A5). One participant, who preferred to maintain the same chat context, continued to use the original *DiaryGPT*. Two authors experienced anomalies when using *InterviewGPT* that led to skipped questions as detailed in Section 3.4. Due to these errors, all authors were provided opportunities to anonymously add or clarify information as part of data analysis and coding.

### 3.3 Data and Analysis

Our diary analysis focused on specific, detailed use cases of GAI and is presented in an amalgamated format to preserve authors’ privacy. The interview analysis centers around patterns of use and broader expositions of themes and is presented as a synthesized discussion with anonymous author identifiers not corresponding to author order (A1-A8).

#### 3.3.1 Diary Analysis.

The team submitted fifty-five diary links with DiaryGPT conversations through the Google Form submission. Diary content was analyzed at the end of the collection period. Submitted diary data included over five pages of written reflections per author(after cleaning the data/removing GPT chat responses). Diary responses ranged from less than a page to responses longer than five pages with supplementary, raw chats over twenty pages in length attached. Some diary links submitted consisted of multiple, consecutive diary entries or covered multiple instances of GAI within a singular entry. Overall, we collected sixty-seven reflections on GAI use for accessibility across our eight authors, averaging around eight reflections per author during the study period. These entries did not represent all GAI use by authors during the study–some authors did not diary about daily, personal use they felt was outside of the scope of accessibility.

The first authors split the diary entries into snippets of several consecutive sentences and independently performed inductive coding on the entire diary dataset [136]. Additional authors met synchronously to discuss codes and then performed a third round of coding on the same data, with each author assigned to a subset. Within their assigned subset of the data, they were instructed to add missing codes and to highlight representative use cases that they found important. The first authors validated the codes for correctness and consistency, consolidating similar codes to reduce the original six hundred and thirty-seven unique codes to one hundred and seven, of which the most common are shown in Table 3. Two additional meetings with authors were held to discuss themes and representative use patterns. Nine use patterns were collaboratively identified and consolidated into higher-level themes with shared attributes. The final set of four themes includes saving time, communicating and conforming, social substitution, and understanding and consolidating information.

Based on these themes and use patterns, we collaboratively developed illustrative vignettes, amalgams of the data we collected. This is a privacy-preserving approach allowing authors to describe shared experiences without outing specific disabilities or identities, similar to that used in prior collaborative autoethnographies [45, 80, 114, 143]. Vignettes each illustrate a concept with one or two different demonstrative examples capturing different types of experiences and are labeled with an anonymously selected name (*e.g.*, Vignette 1: Max and Vignette 2: Rachel). Most of the vignettes represented the experiences of multiple authors. Authors self-assigned the writing of vignettes that personally resonated with their own experiences and contributed their perspectives to shaping the stories presented.

#### 3.3.2 Interview Analysis.

Before analysis, six snippets from interviews that detailed de-anonymizing, task-specific use cases of GAI for accessibility were integrated into the diary responses dataset, resulting in six additional reflections, and analyzed in that context. The interview data analysis proceeded similarly to the diary analysis. Authors also met twice to discuss meaningful quotes and reflect on themes.

### 3.4 Methodological Reflection

In addition to analyzing the data we collected, we used completion data and commentary from authors to reflect on our methodological approach since using a chatbot for interviewing and diarying is relatively novel.

GAI chatbots proved an accessible, anonymous, and convenient medium for data collection. Yet the use of chatbots as a data collection tool had challenges, with the *DiaryGPT* requiring a corrective iteration (described earlier) and four of eight participants experiencing issues with *InterviewGPT*. A4 asked GPT for a list of remaining questions and selected the ones they wanted to complete out of those, leaving a substantial number unanswered. A5, who opted to use voice input GPT to complete the interview, experienced a technical “glitch” and lost all of their progress halfway through the interview. They opted to redo it, completing it through their phone’s text-to-speech functionality. A1, returning to the interview after requesting a break, received a condensed interview with twenty of the fifty-four questions consolidated into two longer questions. Finally, A6 inadvertently caused *InterviewGPT* to skip ahead in the interview by expressing frustration with the repetitive interview questions:

A6: *I feel like I’m repeating my older answers at this point!*

GPT: *I appreciate your patience and understand it might feel a bit repetitive. Let’s shift focus a bit: Over the past few months, how has GenAI impacted access for you?*

The author accidentally “co-opted” the interview, triggering InterviewGPT to skip twenty-two of the fifty-four questions. Many of these outcomes could be typical of any interview or survey, from the lost data to A4’s choice to answer only some questions, and three authors completed the interview in segments, with breaks. Other experiences with InterviewGPT demonstrated properties unique to conducting interviews through GAI, including the modified interview protocol experienced by two authors. InterviewGPT also asked minimal follow-up questions despite the training prompt instructing it to do so (Appendix A), a limitation compared to a skilled, human interviewer. Our analysis method of repeated discussion helped to compensate for this lack of in-the-moment follow-up.

The privacy provided by chatbot journaling is an important advantage of our approach. Unfiltered, honest reflections, combined with our vignette-based synthesis of the data, enabled authors to reflect on their GAI use without fear of being individually identified or stigmatized. As individuals with often-stigmatized conditions [46], this approach, even within our team, allows for “saving face” [85] when discussing vulnerable moments of GAI use and maintaining agency in the presence of institutional legal policies or mandatory reporting statuses [61, 149]. When asked where they would be comfortable disclosing aspects of their GAI use, some authors dissented, “*Not at all comfortable! Thus, the anonymity*” (A3). Others described concerns with sharing their disabled identities or mental/physical health conditions, “*This interview, for instance, is already pushing the boundaries of what I am willing to share [about my conditions] . . . My concern is that a malicious agent can break into OpenAI and extract chat histories*” (A5). Multiple authors only participated with the level of detail that they did due to privacy-preserving considerations in our methodology.

## 4 Results

Four primary themes arose from our analysis of daily GAI use: saving time and mind, communicating and conforming, social substitution, and understanding and consolidating information. In each case we begin by describing the theme, and then illustrate it with one or more vignettes. We also highlight where concepts of stigma, camouflage/masking and intersectional experiences arose and discuss other trade-offs and benefits of GAI use.

### 4.1 Saving Time and Mind

Fluctuating abilities, perception, and symptoms can conflict with normative, inflexible work contexts [18, 90, 117, 124]. For example, neurodivergent people can experience “time blindness” [18, 117] and use specific access strategies for staying focused and task completion [31]. Others experience *crip-time*, at times working slower or on different schedules than their peers [76, 124]. Linked with time is the concept of *spoons*, a term coined in the chronic illness community to describe daily limited availability of mental and physical energy [101]. Authors reported using GAI to save time and mental energy by using GAI to deliver critical tasks on a timeline in Vignette 1: Max, and using GAI to automate non-critical but mentally draining tasks in Vignette 2: Rachel. Through these two vignettes, we present representative cases of GAI use at work and school by novice and expert-level employees, selected because prior research indicates that domain expertise affects both GAI use and satisfaction [15].

Max uses GAI by necessity to save time on high-stakes, critical tasks that need to be delivered within a normative time frame. All authors described using GAI to save time or keep time on their work tasks, “*It helps with my neurodivergence (ADHD) for sure. GAI goes really well [with] my cognitive process and expedites what I want to do, keeps me on track and [keeps] momentum*” (A4). However, three authors describe risks of GAI use, such as over-dependence [25], as concerning, “*generative AI takes away a valuable opportunity to develop troubleshooting and researching skills. And overdependence upon generative AI means the part of the brain responsible for gathering, collating, and interpreting information becomes weaker over time. This is a skill I still want to hold onto so when I use generative AI that is the trade-off*” (A5). Yet, as one author describes, the decision to use GPT sometimes feels compulsory, “*Sometimes, I don’t really choose to use it. It is survival mode, I am tired, slow, and class is starting in 20 minutes. . . I really need to know what that paper means*” (A3). For these authors, GAI is a way to camouflage their lack of conformance to normative timelines, sometimes at the expense of their long-term growth.

Three authors recount using GAI to conserve mental energy (brain-spoons), which is “*randomly there, or not there. [Thanks to GAI] I can still get things done when it’s not there, which I appreciate*” (A6). Utilizing GAI to conserve mental energy helps ensure that “*I can focus my time, effort and interest on things that are more productive and exciting for me*” (A5). Yet the use of GAI for automating tasks comes with risks. Multiple authors acknowledge subpar outputs as being inherent to GAI use, “*I kind of like that because it forces me to check it every time I use it. If it always got it right, it would be less of a tool and more of a substitution*” (A4). However, such validation itself requires spoons, potentially adding to the stigma authors experience “*. . . if I don’t have the energy to validate, then I could make myself look like a clown if I bring info to others. Usually I try to validate my work though, but I have taken risks and not done so*” (A3). We see here how masking is deeply intertwined with energy resources [71].

Vignette 1: Max and Vignette 2: Rachel both illustrate the value of GAI for conserving time and mental energy. However, in Vignette 1: Max faces work pressures that force him to forgo opportunities for growth and learning [25, 88]. In contrast, in Vignette 2: Rachel uses GAI for automating menial tasks due to the internal need to conserve mental energy. In both cases, verification is necessary but requires work that is not always accessible for the person doing the verifying [45]. Authors illustrated by Max are “blocked” by erroneous outputs, while those Rachel illustrates more easily correct and adjust for errors when they have the spoons to do so in time. All of the authors understand the limitations of GAI, such as erroneous outputs and over-reliance [24]; it is normative expectations and external pressures, such as the need to meet deadlines or keep up with a fast-paced work culture, that force some authors to use GAI in risky ways despite those limitations.

### 4.2 Communicating and conforming

Social acceptance and associated normative standards are a frequent cause of both neurodivergent masking [3, 13, 106] and of “code-switching” for people with different linguistic and cultural identities [6, 157]. Both masking and code-switching have serious negative ramifications for mental health and internal concepts of self [119], and experiencing “race and neurodiversity together is exhausting,” [84, p. 1309]) with normative expectations of the White gaze intensified by neurodiversity [84]. The following three reflections highlight uses of GAI to conform : code-switching language as a mechanism for fitting in (Vignette 3: Isaiah), matching normative standards of emotional expression (Vignette 4: Sanya), and preemptively diffusing social stressors (Vignette 5: Sam).

Isaiah’s story captures the conflict between identity and normative professional standards, which GAI cannot resolve: “*the writing reduces my tone or tries too hard to be formal, making the writing sound weird*” (A4). As one author reflects, “*sometimes accessibility comes at the cost of my other identity facets such as culture*” (A8). This erasure of identity further marginalizes authors with diverse identities. “*There is a tradeoff between acquiring access and erasing my cultural/linguistic identity specifically in the case of interpersonal communication. So this begs the question of ‘is this really access’?*” (A8). In contrast, Two white authors describe positive experiences collaborating with and learning from GAI: “*GAI has also taught me if my communication is unclear. Because then it will absolutely botch a rephrasing and I always have to go back and reword it and think about what I’m saying. So I am learning through GenAI, slowly*” (A3). The (White) authors’ reports of their experiences center on clarity of communication rather than masking identity.

Sanya’s anecdote highlights how GAI use supports both *emotional intelligence* [142] and cultural adaptation in expression and communication– two needs that for authors are deeply intertwined. These aspects of communication cannot be easily disentangled for authors represented, and GAI does not differentiate between them: “*I never thought of myself as very great at English, and not great at expressing my thoughts. It’s hard to find the right words sometimes, but ChatGPT seems to be able to convey its thoughts seamlessly, in a way that flows perfectly*” (A7). Despite GAI reinforcing cultural conformance, authors viewed its support in facilitating emotional expression and communication positively regardless of their racial and cultural background, both in terms of the quality of the results and the positive learning experiences provided: “*I think my interactions with people in general have become more skillful*” (A5). Although authors are still forced to conform to normative expectations, the risks authors reported were less centered on internal costs such as identity erasure and more about failed camouflage. “*My friends sometime notice. . . some responses do not sound like me at all*” (A2). Authors described consequences such as peers being upset by their use of AI and shaming: “*some people tease me about my use of GenAI, or have ethical judgment to make about it*” (A3). In such cases, the goal of the GAI use– to further social connection–results in the opposite outcome, rejection.

Multiple authors use GAI to pre-emptively avoid social stressors, “*sometimes I use GenAI when I am in argument with someone. I use GenAI to minimize the use of mental energy*” (A2). Planning and strategizing with GAI helps authors navigate difficult social situations and avoid adverse experiences, such as freezing up, brain fog, and fatigue. Their GAI use reinforces masking by strategically molding authors to conform to stressful social settings to avoid negative consequences. Concerns authors raised had to do with access to correct and complete data for better support, and privacy.

The anecdotes in this section emphasize the tensions authors face when using GAI to navigate social spaces. GAI helps Vignette 3: Isaiah to learn about cases where communication is unclear, Vignette 4: Sanya to achieve more authentic self-expression, and Vignette 5: Sam to mitigate fatigue and avoid freezing and brain fog. While each of these cases involves conforming to normative expectations, we see how interactions that focus on collaboration and learning are mostly positive. In contrast, experiences of inauthenticity and erasure, which differentially impacted non-White authors, were harmful and inaccessible. In the words of A8, “*There shouldn’t have to be additional prompting for GenAI to be more representative.*”

### 4.3 Substituting GAI for people to reduce social costs

Neurodivergent people face peer rejection and loneliness [67, 129, 141], which can affect mental health and lead to poor outcomes [23]. Yet, accessibility technologies often neglect access needs such as emotional regulation [152], and alternatives like social support can be unavailable or inadequate. We illustrate two examples of neurodivergent people who leverage GAI for social support.

In Raine’s vignette, GAI is a preferred alternative to peer connection, even when a peer is readily available. In the words of A3, “*there are so many things I can ask GenAI about, and it won’t judge me by default. I love that. I love being able to share things, ask things, and not have to worry about what people will think. . . People aren’t always patient or kind if you ask an ‘obvious’ or repetitive question, GenAI is always kind and eager to answer*” (A3). Authors describe GAI as having multiple advantages over peers, including reduced temporal and cognitive costs, lack of pressure to meet normative expectations, and lack of judgment.

Factors such as perceived burdensomeness impact authors’ interactions with others, “*I have dumb questions at work. I don’t want to bother senior [coworkers] with questions I could google, so I save them for ChatGPT*” (A7), encouraging GAI-based substitutions. These concerns extend to even emotional areas where peer support could be beneficial, with multiple authors stating “*. . . other people don’t want to be burdened down by your troubles. I know I am not inconveniencing anyone when I chat with ChatGPT*” (A5). Authors who used GAI in this way valued its support, but varied in their opinions of its quality. For example, A7 stated “*Mentors are so important in every step of your life, and ChatGPT, when used properly, honestly has more knowledge than any mentor out there in real life*” (A7) while A1 acknowledged “*[GAI] improved [my mental health] by allowing me to feel less anxious knowing that I can always have access to feedback and reassurance. . . though there is still a large disconnect in terms of how helpful it is to get reassurance from ChatGPT in its current state as opposed to a real human/mentor*” (A1). Perhaps this reflects differences in the availability of human mentors in different authors’ lives. In the end, the high costs of meeting social needs, due to being judged for the timing, frequency, and content of requests, is an unacceptable price to pay for these authors, making GAI a necessary, and sometimes good, alternative.

Social barriers faced by neurodivergent people include ostracization, judgment, and rejection [67, 129, 141]. When GAI is substituted for social interaction, it eliminates these barriers, as well as the need to mask, eliminating significant negative costs for these interactions. It is thus no surprise that authors valued GAI’s benefits in this context. GAI for mentorship tasks outside of emotional support or reassurance can introduce risks such as producing misinformation or bad advice [139]. Furthermore, using GAI for emotional support raises concerns about fostering over-dependence [63]. Despite these risks, authors’ experiences in using GAI for emotional and peer support were largely positive and helpful, aligning with existing findings on chatbot use for mental health support [95].

### 4.4 Help with understanding and consolidating information

Information access has been highlighted as a critical need, described as a “*fundamental freedom*” and key to “*building inclusive knowledge societies*” by UNESCO [144]. Yet documents with critical information, such as legal and medical documents, remain inaccessible for many, including people with cognitive disabilities or impairments [145]. These reflections demonstrate authors using GAI to make complex, domain-specific information more accessible.

In Liam’s vignette, GAI provided valuable access to a “wall of text” full of legal jargon. Multiple authors reflected on the importance of both summaries, and conversation, for their access needs. Although “*Generative AI has made certain tasks require fewer spoons*” (A5) for some authors, others report that the need for prompt engineering and iterative feedback can be a barrier. “*I think my ADHD makes it hard to stay engaged with ChatGPT, and so I often will give up on using ChatGPT for something if it takes too much prompt engineering, iterative feedback, etc.*” (A1). Additionally, when GPT provides inaccurate or overly detailed information, it can divert users into unrelated areas of research, leading to a loss of focus and interest. “*Sometimes GenAI provides parrot information or wrong information. This causes me to do more research that is not related to task and causing me to dig another rabbit holes, and often losing interest on the work I was doing*” (A2). Even requests for concise answers can result in overly lengthy responses, adding to the cognitive load and further complicating the research process: “*I always ask for a short and concise response, but GPT just loves essays*” (A5). This frustrating experience can result in wasted energy and time, “*I usually move on after following up with a few clarifying questions. If GenAI still fails to prompt correct response, I just go on websites or look for related papers. This is the same way how I used to consume knowledge*” (A2). Iterative information access can be a great tool for these authors to delve into and discover information, but can also present access barriers when arriving at a satisfactory output is too cognitively taxing.

Multiple authors “*use [GAI] for text simplification to make information more understandable*” (A4), particularly in times of cognitive impairment such as brain fog or illness: “*My health took a turn for the worse and made things like summarizing papers or getting concisely-written explanations more needed*” (A3). GAI improved accessibility by “*simplifying complex medical terms. . . answering questions more straightforward[ly], summarizing long documents and clarification*” (A2). Authors describe how GAI was helpful for rapidly simplifying information during times of impaired cognition. Yet, in these times, they depend on GAI to deliver accurate outputs, “*It’s very important that [GAI] gets it right. It’s the whole point - if I can’t trust the information, it’s no better than me asking questions to my dumbass friend who believes in every conspiracy theory out there*” (A7). Authors emphatically state that receiving correct information from GAI is critical for its usefulness in making information accessible, “*for generative AI to play a role in people’s lives, it cannot feed us the wrong information. doing so would cause us to lose trust, and would hurt its usefulness*” (A5). Despite this strong need, authors acknowledge that GAI in its current state is prone to misinformation, desiring not only accuracy but also self-reflection. “*If GAI is unsure of an answer, mentioning that the answer may be incorrect and offering other sources or ways to find the right answer will help users to have less confusion*” (A2). The tradeoffs participants must make under the pressure created on the one hand by ableist systems that may also not give them accurate answers [70], and on the other by their own disability needs, forces them to select the best bad option for where and how to get information.

Both of these vignettes illustrate the value of GAI for information access. Vignette 8: Liam is accessing domain-specific information through back-and-forth conversations, which helps him to gain a deeper understanding of a document but can add to the cognitive burden or lead to distraction. In contrast, Vignette 9: Kai is interacting with GAI *because of* cognitive difficulties, such as during exacerbations of illness. Kai faces risks such as acting on erroneous output or misinformation due to lack of mental energy to validate outputs. Interestingly, authors represented in this theme do not emphasize challenges relating to stigma, masking, or intersectional identities outside of the self-stigma around seeking help and English language comprehension described as motivating factors for GAI use in Kai. Perhaps this is because these examples arose from situations that required cognitive access but did not include the same sorts of normative and time pressures represented in earlier themes.

## 5 Discussion

The insights from our autoethnography demonstrate the innovative and powerful ways neurodivergent people use GAI for accessibility. Our findings align with assistive technology needs identified by Spiel et al. [132], giving us confidence in the representativeness of our results. Spiel et al. identified technology opportunities in helping neurodivergent individuals meet societal norms, supplementing their existing coping strategies [132]. Authors use GAI for modulating communication to match neurotypical standards, such as code-switching to meet academic language expectations shown in Vignette 3: Isaiah, or emulating cultural norms for emotional expression in Vignette 4: Sanya. Our data also shows uses of GAI to self-manage emotional regulation– an area of need described by prior work [153], such as the use of GAI as a companion for processing emotion in Vignette 6: Raine, and as both a tool for on-demand emotional support and motivating self-action in Vignette 7: Alex. While people-based solutions such as body doubling [32] or peer support [153] also help neurodivergent people to meet emotional regulation or self-management needs, the lack of peer availability or unconventional, “crip time” working hours [76], and fear of social judgment or rejection, make GAI an alternative that fosters agency and flexibility.

Our findings also present a nuanced picture of GAI risks when used for accessibility, with concerns around privacy, built-in biases and lack of representation, and errors. The authors are aware of these risks and still use GAI to meet their access needs, not because it is an ideal technical solution, but because it is sometimes the best tool available to navigate the societal, structural, and systemic barriers they face. Through our discussion, we unpack these dynamics and consider how GAI both empowers “power users” and presents new concerns.

### 5.1 GAI enables autonomy and privacy when sharing neurodivergent experiences

Stigma remains a pervasive issue affecting neurodivergent individuals’ relationship with their disabled identity and interactions with accessibility tools, including GAI. Stigma is not just theoretical; it is deeply personal, shaping the authors’ professional, academic, and personal experiences and influencing the design and interpretation of this ethnography. Our bespoke tools – InterviewGPT and DiaryGPT – help to address the issue of stigma while providing authentic, “un-masked” reflections of our GAI use. These tools give us autonomy in disclosing meaningful yet potentially-harmful aspects of how our disabilities impact our work and lives, and allow us to find a sense of relatedness in how we use GAI despite inherent power differentials. By employing and reflecting on the methodological use of InterviewGPT and DiaryGPT [121], our subjectivity as both creators and users of these tools allows us to critically reflect their benefits, shortcomings, errors, and resulting access conflicts. We encourage future work to embrace neurodivergent participants as stakeholders in their own data-sharing experiences [151], and to center and adjust to participants’ needs [91] rather than enforcing rigid data collection design, allowing for agency and control over what and how experiences are shared.

### 5.2 GAI use for sensitive contexts is an ongoing reality

The use of GAI by neurodivergent and disabled individuals to meet needs in sensitive contexts precedes this autoethnography. As described in the introduction, deployed GAI disability and mental health tools are actively in use. This reflects a pressing truth: despite GAI’s flaws, individuals are already using GAI for sensitive contexts, often as a necessity, because no other tools or systems adequately meet their needs. Our study’s authors use GAI in sensitive domains, and do so despite being aware of common errors and risks. Authors use GAI for emotional reflection and support in vulnerable moments in Vignette 6: Raine and Vignette 7: Alex and they use GAI in scenarios with real-world consequences, such as navigating legal (Vignette 8: Liam) and medical information (Vignette 9: Kai). The “power users” in this study were not ignorant of GAI issues such as built-in biases, misinformation, and overdependence. They made informed decisions to use these tools because the potential harms they could encounter from GAI were less threatening than consequences such as facing real-world, systemic harms such as marginalization or consequences for failing to conform to normative standards.

However, authors were especially vulnerable to GAI risks when dealing with fatigue, cognitive overload, or limited energy (“spoons”). GAI outputs require validation, which authors could not always manage accessibly, putting them at risk of producing erroneous or low-quality work when not able to provide the required human oversight [24, 45]. Moreover, authors expressed concerns regarding the potential negative impact of GAI on their learning and development, highlighting its risk of inhibiting deeper understanding and meta-cognitive skills [88].

Given these concerns, future research should not focus on deciding when and how neurodivergent people should use GAI– they are deciding that for themselves. Instead, research should directly address neurodivergent and disabled people’s needs for improving accessibility of GAI tools, such as incorporating built-in validation [45], while deferring to their agency and autonomy in how and why they use GAI, acknowledging them as experts in navigating and mitigating both social and technological harms.

### 5.3 Intersectional identities impact GAI’s utility as an accessibility tool

Future research must investigate how intersecting identities – particularly race, culture, and linguistic diversity – shape the experiences of neurodivergent people in interacting with GAI. Our findings illustrate that code-switching and masking needs differ across sociocultural contexts and impact satisfaction with GAI use. While authors engage in code-switching to navigate professional settings and reduce marginalization in such environments, they experience a profound loss of cultural and linguistic identity (Vignette 3: Isaiah) in these modified communications. The additional effort required to make GAI authentically represent them in these contexts further diminishes the accessibility of the experience. In contrast, when authors from immigrant backgrounds use GAI to aid with emotional intelligence and expression (Vignette 4: Sanya), they appreciate the resulting linguistic modifications. These divergent experiences require future investigation to understand, reconcile, and surface the value of including both theoretical understandings, and participants, representative of varied cultures, backgrounds, and experiences [52]. Understanding these differences is essential to designing tools that respect and preserve identity while supporting access.

### 5.4 GAI “power users” seek agency in GAI use, and in life

Our data repeatedly shows how the most successful uses of GAI for access puts control over when and how access needs are met squarely in the hands of the user, giving them increased agency rather than “training” them to conform. As described by Williams and Park, self-directed flexibility and control are important accessibility factors [153]. In our study, interacting with GAI provided authors with agency—not only by granting them control and flexibility [153] in using the tool itself but also by empowering them in their broader lives, where ableism and stigma often strip away autonomy and relatedness [29]. Our vignettes illustrate times authors lack agency in their lives due to external forces, and turn to GAI to facilitate their own empowering, agency-preserving experiences. Vignette 5: Sam faces systemic dismissiveness in a medical setting, which drives them to adapt their language through GAI to ensure equitable care. Vignette 6: Raine and Vignette 7: Alex attempt to fill a dearth of relatedness and use GAI to access emotional support that is unavailable or costly– but needed. Vignette 2: Rachel maintains autonomy by completing workplace tasks at a desired normative cadence despite brain fog, ensuring mental energy is conserved for other, needed tasks. None of the authors in these vignettes are seemingly positioned to drive systemic improvements in their environments or social settings. Instead, they use GAI to carve out their own areas of control and flexibility in systems too rigid to accommodate their needs. For these authors, GAI use represents a tool for reclaiming autonomy, relatedness, and competence within environments that often deny them these essential elements of self-determination [29]. Future research should not only ask whether GAI provides individuals with autonomy in modular interactions, but also whether it empowers users to reshape their interactions in broader environments and reclaim agency in contexts that systematically deny it.

In summary, while GAI offers novel and powerful (if also flawed) opportunities for accessibility for neurodivergent people, its use also reflects the systemic barriers neurodivergent people encounter daily— necessitating its use. Technology can be a powerful aid in self-defining what access means, but true access never lies only in the hands of the disabled person. Ableist and racist individuals, policies, and societal structures must also be addressed. The detrimental impact of ongoing epistemic injustices against neurodivergent people cannot be underestimated [39, 72, 96, 159], nor cannot it be solved only by GAI.

## 6 Conclusion and Future Work

Our autoethnographic exploration into the “power use” of generative AI (GAI) by neurodivergent researchers and technologists reveals both its potential benefits and harms. Through diaries and interviews, our authors’ reflections illustrate how GAI use fosters agency and access in when and how they work, communicate, seek emotional support, and find information, while meeting historically unaddressed access needs such as emotional regulation [153] and conforming to normative demands [132]. In academia and the workplace, where neurodivergence and disability remain stigmatized, GAI use is often not just a tool for access but a mechanism for survival—a means of masking, conforming, and mitigating bias in spaces that remain resistant to structural change. Yet GAI use places the burden of adaptation onto “power users” themselves, reinforcing normative expectations rather than challenging the systems necessitating the adaptions. The risks of using GAI, such as bias and lack of self-representation, reduced learning and skill development, producing low-quality, inaccurate work, or harm to wellbeing [139] must be addressed to ensure that GAI serves as a genuine tool for neurodivergent accessibility rather than an instrument of conformity.

Future works should also explore the use of GAI by a broader sample of neurodivergent people, including those with a wide range of educational, cultural, and global backgrounds. It is imperative that future research on neurodivergent people’s use of GAI considers not just the use of these tools to address specific accessibility needs, but how GAI and other tools can address the broader societal and environmental forces influencing or necessitating its use, instead of reinforcing them.

## Figures and Tables

**Table 1:** GAI use history and frequency

	First encounter	Frequency of Use
**A1**	ChatGPT 1 year ago (or slightly less)	“*When working/in school, I often interact with ChatGPT at least once a day*”
**A2**	GPT 3.5 on Dec. 12, 2022	“*When I am studying or doing a big project approximately 5–7 hours / day... just using GPT for a personal use, it would be less than 1 hour / day*”
**A3**	DALL-E	“*You know, it varies. I would say anywhere from half an hour to several hours depending on the day or task*”
**A4**	January 2023	“*Probably ~5 hours a day*”
**A5**	“*Early days when DALL-E was still research access only*”	“*… ChatGPT and similar tools, then perhaps half an hour a day. But if you include the usage of [other LLM tools], then that number jumps to hours*”
**A6**	ChatGPT [when] it had just come out	“*Not much. Maybe half an hour or less per day*”
**A7**	Roughly December of 2022	“*I’d say on a normal day, maybe 5–10 minutes. There are some days (some weekends) when I might actually not use it at all. Then there are days that I go back and forth with chatgpt for an hour*”
**A8**	ChatGPT	“*Its hard to estimate, but a couple hours*”

**Table 2:** Self-described Identities Represented. Counts are not shown (e.g. multiple authors identified as “Woman”)

**Genders**	Male; Woman; Mostly Female; None
**Race / Ethnicity**	White/Caucasian; Chinese/Chinese American; Asian; Black/African American
**Languages / Dialects**	English; American Sign Language; African American English; Chinese; Korean; German; Russian
**Immigration History**	1st Generation; 2nd Generation; Generational American

**Table 3:** Frequently Occurring Codes from Diary Entries. Each column shows a list containing: code (# times found)

Major Use Cases	Use Context	Descriptors of Use	Interactions with Output	Tools Used
Communication (55) Writing (51) Info (46) Learning (34) Coding (29) Social-Skills (24) Revise (23) Prep (20) Save-Time (19) Social-Replace (18)	Academic (31) Medical (29) Work (26) Mental-Anxiety (18) Cognitive-Issues (17)	Good Experience (41) Bad Experience (22) Mixed Experience (14)	Prompt Engineering (18) Inauthentic voice (14) Fixed outputs (12) Re-prompted (11) Error in output (6)	GPT (102) Unspecified GAI (11) CoPilot (7) Gemini (2)

## Vignette 1: Max, crip-time adjustment, (amalgam of 4 reflections)

Max, who has ADHD and migraines, uses ChatGPT to generate code and learn complex technical concepts for their junior research engineering job because their symptoms have been making them “*. . . kind of slow at work so I’ve been prioritizing finishing my [tasks]. . . I just copy paste [the code] and hope [GPT] understands what’s wrong.*” They need to learn a difficult software concept before their daily check-in with their team lead, and turn to GPT, “*I have low cognitive abilities today and had a migraine yesterday so reading large text blocks is kind of uncomfortable still so I didn’t have to read Wikipedia which was nice and just got a tailored answer explaining it in an understandable way.*”

The team lead, happy with his learning, assigns Max a new, complex AR/VR project to start immediately and Max uses ChatGPT “*to help me with coding on my Vision Pro app.*” However, the novelty of the technology leads to frustrating results: “*I used ChatGPT and Gemini, and both of them didn’t know what they were doing. They would give me code, I would try it out, and there would be errors, compilation errors. And. . . I just didn’t know how to fix most of them.*

I wasn’t sure what to do, because I usually always go to Generative AI to learn things.” However, Max recognizes tradeoffs in using GPT to speed up learning: “[GPT] helps me keep moving and make progress in everything I do, my busy work, my communication, my research. And maybe I have relied on it so long that sometimes I don’t feel like I can be successful without it. . . Personal growth and learning is 100x more valuable than finishing this ticket one day earlier. I’m realizing it more now. . . ”

## Vignette 2: Rachel, brain-spoon management (amalgam of 4 reflections)

Rachel, who is a neurodivergent technologist, uses CoPilot and GPT to automate “*repetitive tasks like defining enums*” or generating bibtex entries. These tasks are “*time consuming and [use] up brain-spoons.*”

Rachel avoids using GAI for critical tasks, preferring it as a “*a form of advanced autocomplete*” for menial, yet draining, tasks. It also serves as a memory aid–rather than scouring a document, she “*ask[s] ChatGPT to find a word. . . after many attempts it finally gave it to me.*” She has to iteratively provide more context for GPT to succeed. Her code generation similarly requires coaching CoPilot, “*I have an idea of what I want the code to look like. . . [when CoPilot’s] suggestion is of poor quality, I will begin writing what I think is the proper solution, and see if copilot catches on.*” She notes that “*the prompt is very important much like communication with a human.*” Rachel’s level of experience makes her adept at catching errors produced by GAI, and she always checks its work. For example, when reading an (overly “saccharine”) summary of a meeting transcript, she requests “*a list of todos (which it didn’t provide by default, another not great choice).*” The results are still subpar, she “*noticed a lot of redundancy in them and cut some text before sending.*” Despite constantly needing to validate or fix outputs, Rachel continues to use GAI for work, appreciating the mental energy it saves her overall.

## Vignette 3: Isaiah, code-switching, (amalgam of 7 reflections)

Isaiah is neurodivergent, has social anxiety, and was raised in a multilingual household. English is their second language. Isaiah often feels pressured to adapt their language to the “appropriate” and “professional” language expectations of academia. They note, “*[GAI] helps me feel like I fit in because I can conform to typical academic styles.*”

Recently, Isaiah used GAI to help them craft a professional email to a new professor they wanted to collaborate with, but “*my anxiety was delaying me in responding to the email, so I used GPT to help me make sure the wording and flow made sense and to make sure the tone was professional.*” Isaiah trusted the GAI because “*GenAI is trained on [data containing] defaults to what is considered to be the ‘standard’ and what is deemed as ‘appropriate’ in professional and academic spaces. So, I figured it can help to make sure my writing aligns with this.*” However, the email lacked personality and authenticity: “*it definitely took away my entire voice and so I have to tweak it many times to get it to be a proper mix of me and professionalness.*” They add, “*sometimes, I have to tell it to shift its tone in different directions because I can tell when it’s trying too hard.*”

Later that day, Isaiah used GAI to write an informal text message to a friend they hadn’t spoken to in a while. However, they were dissatisfied with the output because “*it does not preserve my identity.*” As a multilingual disabled person, Isaiah reflects that “*there is an interesting struggle that comes with GAI addressing my access needs because at times this assistance comes at the expense of erasing other important identities to me such as cultural nuances and dialectal variations.*”

## Vignette 4: Sanya, emotional expression, (amalgam of 3 reflections)

Sanya has difficulties expressing emotions, and as an immigrant with different linguistic norms, he was raised with a more blunt, concise communication style than his peers. Sanya uses GAI to rephrase his written communications, noting that “*it can help me make sure I am using sensitive and appropriate language since I am not really sensitive and have no idea when I am upsetting someone accidentally.*”

He uses GPT to rephrase a metaphor to make it less likely to offend a peer, describing how “*I didn’t want to use the metaphor ‘running around like a headless chicken’ because the person owns chickens and I thought that may be potentially offensive or socially inappropriate. GPT gave me alternate metaphors that captured the same imagery, and actually explained each metaphor to me.*”

Later, Sanya finds himself in a tough spot, at risk of losing his plant collection, “*[I am] very sad and . . . fear for the fate of my plants.*” Sanya needs to write an emotional post to get help from his community, “*I’m not good at writing these types of posts - I rarely make them.*” So he uses GAI: “*I used GAI for writing a compelling post on Facebook. . . GPT was able to instill more emotion into my post and the post received a lot of attention, support, and interest. I don’t think I could’ve done that myself.*” Sanya notes that GPT could identify his emotions better than he could, “*Sometimes I don’t even know what emotion exactly I am feeling. . . sometimes it will pick words that better describe my insides.*”

## Vignette 5: Sam, diffusing social stressors, (amalgam of 4 reflections)

Sam is a college student with social anxiety and chronic illness who finds verbal discussion particularly overwhelming– causing Sam to freeze up or experience brain fog. For Sam, stressful social encounters have consequences, such as flare-ups of illness. Sam uses “*GenAI to support me with in-person communication. . . for discussion-based classes, it’s helpful for me to come prepared with talking points. . . if I have an idea of what the discussion will be about.*” Sam shared that they “*start with writing out talking points myself and then ask GenAI to check and proofread [them].*” Sam feels more prepared for different directions a conversation might take, stating “*sometimes it helps me navigate [an] upcoming conversation by talking through additional ideas beforehand that I did not initially consider.*” Sam asks GAI to provide these talking points in a bullet-pointed format “*because it feels quick and easy to reference during an actual conversation.*” For Sam, these pre-planned talking points reduce the stress of social interactions in the classroom, making them, at times, more manageable experiences.

Later that day, Sam is anxious for an upcoming doctor’s appointment, expecting to have their health concerns dismissed, a common concern for those navigating chronic illness [33, 70]. To prepare for this, Sam instructs GPT to play the role of their doctor, “*mimic[ing] a human conversation*”, because “*[simulating] a human conversation. . . exercises the parts of the brain that get triggered by social anxiety.*” Sam also asks GPT for help “*dealing with negative feedback. . . logical fallacies from authority figures*” that might be hard to respond to under stress, and to find the correct jargon to discuss symptoms. “*I realized I am using wrong jargon and doctors don’t take me seriously. GPT told me how to rephrase my words into the jargon doctors understand.*” With this advice from GAI, they felt prepared to handle the upcoming interaction, stating that “*these strategies help diffuse the situation while not requiring excessive cognitive effort on my part.*” The result was a success: “*[GAI] gave me some good health advice that I took to a doctor and then the doctor used to get me actual medical care. I think it also reiterated what I told it in more clear language, which I used with the doctor, and the doctor took me seriously.*”

## Vignette 6: Raine, upgrading peer support, (amalgam of 3 reflections)

Raine is a neurodivergent student who utilizes ChatGPT to discuss their feelings during their emotional lows, reflecting that “*as someone in academia, it’s difficult to talk to friends or advisors about my low times because of mandatory reporting statuses and. . . a regular search engine can be difficult because there’s so many blocked things to search. . . and [it] automatically assumes you need help. I don’t need help.*” Raine recognizes that peer support is available but would not meet their needs as well as GPT, which was “*not judgmental about how anxious I was, which was nice.*” GAI also allows Raine to avoid unwanted interventions, “*I’m a very self aware individual, but sometimes I want to be able to talk about my lows without it being a call for help or an excuse for someone to get me help that’s not needed when sometimes I just want the ability to talk. . . GPT just let me vent in peace.*”

As Raine’s low lifts, Raine uses ChatGPT as a companion for self-reflection. “*After I took LSD and had ego death, I became really interested in psychology and how the neural pathways that I formed [in my past] shaped myself to be who I am today.*” Raine reflected on their discussion with GPT, stating “*I remember every single response it gave back to me being a new insight about myself. It was mindblowing.*” Raine prefers having these kinds of philosophical discussions with a chatbot rather than peers because “*. . . the friends I have in real life–they can’t keep up as well.*”

## Vignette 7: Alex, creating support in absence, (amalgam of 4 reflections)

Alex is a disabled student with a severe anxiety disorder who regularly “*turn[s] to GPT [to discuss] trivial problems rather than talking to humans first.*” Alex has been told he is annoying in the past by others, so he has stopped asking others for help, “*I. . . don’t feel like I am being a burden when I ask GPT for help as opposed to a human.*” He turned to GPT when he “*[was] very anxious and upset about a paper review that I got*” to avoid “*having to dump my negative emotions on others.*” Although he would trust reassurance from a peer or advisor more, he uses GAI knowing “*I would annoy them less and hopefully make their day less stressful. . . One of the biggest coping mechanisms that I have found to help me push past my task paralysis is reassurance from others that I trust or knowing that I can get reassurance or support from them, like a mentor telling me that I’m on the right track.*”

Late that evening, Alex’s anxiety subsides, and he finally has the energy to write. Knowing his advisor is not available, Alex engages GAI: “*GPT gave me good advice on how to be polite and write a rebuttal.*” Alex appreciates how GAI “*lets me bounce ideas around in a discrete environment, and without sucking up the time of others.*” Yet he wishes he were able to do so with his peers, “*I know I am sacrificing the opportunity for social connection and building bonds with others etc. etc. to get anxiety help from an AI automation*”, but he is unable to overcome the fear of burdening others.

## Vignette 8: Liam, interactive information access, ( amalgam of 6 reflections)

Liam, who has ADHD, is going through a difficult time in his life dealing with legal issues and using GAI to help summarize a legal notice he received. He reflects that, as “*an individual with ADHD who finds it difficult to sit through large walls of legal jargon, GPT’s summary was a godsend.*” Liam explains that “*legalese is very difficult to interpret without generative AI.*” In addition to summarizing, GAI can respond to many follow-up questions on details he is unsure about. The back-and-forth with GPT is helpful because “*reading all the related laws and articles sometimes is confusing. . . by using GAI, I was able to learn what I need to do.*” However, as he finalizes a response to the letter, he notes “ *ChatGPT did not give me an answer that I felt confident enough to take at face value. . . I ended up calling an actual lawyer in the real world.*”

Following this experience, Liam decided to consult GAI to help a friend with legal matters: “*I needed to rapidly find information about what reasonable accommodations translates to in many settings but did not have a good idea of the best search terms. . . I asked ChatGPT instead.*” This time, GAI was less accurate: “*[it] found 3 possible answers, one of which was helpful, but that allowed me to find a new phrase and use it in my follow up question.*” This back-and-forth helped Liam uncover a helpful legal case, “*. . . so between the two queries I had two links to supporting information that I could then summarize and share with the person I needed to provide information to.*”

## Vignette 9: Kai, simplify info to ease understanding, (amalgam of 6 reflections)

Kai has an illness causing frequent brain fog, and self-stigma around seeking help [58] due to prior poor experiences with doctors. Kai uses GAI to get health information when experiencing brain fog, such as asking “*numerous questions about surgery and my rehabilitation protocol.*” Kai experiences a “*weird health symptom*” and uses “*GPT to ask about it. . . I have ADHD and don’t want to read like 100 google search results because they always present things in these long annoying lists.*” Kai uses GAI to simplify and summarize medical information “*because I didn’t have the brain power to read lots and lots of potentially irrelevant search results to find the information.*” Once Kai “*. . . found the right journal article to read, I also used it to translate medical terms that were unfamiliar to me.*” With an idea of what his symptom could be, Kai asks for more advice in an easy-to-digest format, “*GPT gave me some suggestions on what could be causing it, and then some tips like medications on how to deal with it.*” In these periods of brain fog, Kai appreciates “*how convenient [seeking health info through GAI] was, its ability to ease complex medical jargon for a non-native English speaker.*”

## Footnotes

1 DiaryGPT, A GPT-based diary interface built through OpenAIGPTs (https://openai.com/index/introducing-gpts/)

2 InterviewGPT, A GPT-based interview interface built through OpenAIGPTs (https://openai.com/index/introducing-gpts/)

## A DiaryGPT contents of Questions.txt

### Prompt:

DiaryGPT: This GPT will ask questions from Questions.txt, one at a time. It will wait for responses before asking the next. It will never send walls of text regarding an experience. If appropriate, it will ask relevant follow-up questions to learn more about how GPT-4 was used to address an access need. You will never sound like a condescending therapist. Talk like a friendly person.

### List of Questions:

- What did you use GPT for today?
- What access, disability, or mental health need did GPT help with today?
- Why did you think GPT would be helpful for this?
- Did it make any mistakes, and if so how did you catch them?
- Did you quote it directly in any communication, rephrase it, or something else? if other, what?

## B InterviewGPT contents of Interview.txt

1. “Thank you for participating in our research study! I will now ask you several questions about your experience over the last few months. This interview will take approximately 45 minutes. There are no right or wrong answers to these questions; we are interested in knowing what you really think. You may refuse to answer any questions that you are uncomfortable answering. You may stop this interview at any time or skip questions. The research team will protect any information you provide, which will be used only for research purposes and not reveal who you are. Are you ready”?
2. When was your first encounter with GenAI? What tool or software was it?
3. Why did you initially decide to use it? What were your expectations?
4. What GenAI tools have you been using since then?
5. “Are there parts of your life (i.e. work, school, personal) that you use GenAI tools for? In these parts of your life, what kinds of tasks do you mainly use GenAI tools for”?
6. How does GenAI succeed at performing these tasks?
7. Have you experienced any problems while using GenAI for these tasks?
8. Has there been anything you found especially delightful while using GenAI? What have you liked about your experience with GenAI?
9. Does GenAI remind you of any other app, technology, or non-digital experience you’ve used/had in the past?
10. Why do you choose to use GenAI instead of any of these alternatives (i.e. app, technology, non-digital experience)?
11. How do you choose what tasks or purposes to use GenAI for?
12. Have you used GenAI as a tool to achieve specific goals?
13. Are you satisfied with your performance towards achieving these goals?
14. If you were to continue using GenAI, do you think you would keep using it to achieve the same goals, or at what point or under what circumstances do you think you would change your usage of GenAI?
15. Over the past few months, how has GenAI impacted access for you?
16. Has GenAI changed accessibility of specific content (e.g. documents, written materials) or tasks you perform?
17. In what ways, if any, has GenAI created barriers to accessibility for you?
18. In what ways, if any, has GenAI improved accessibility for you?
19. Has GenAI impacted your mood or mental state? If so, how?
20. What moods or mental states are you typically in when engaging with GenAI?
21. Has GenAI impacted your health or physical wellbeing? If so, how?
22. What physical states are you typically in when engaging with GenAI?
23. How much time approximately in a day do you spend engaging with GenAI tools?
24. How much time approximately do you think engaging with GenAI either adds to or subtracts from time you would usually spend on the same tasks?
25. How does using GenAI impact your spoons? (If you are unfamiliar, see spoon theory)
26. One theme from the diaries was the use of GenAI to conserve mental energy. Do you use GenAI for this purpose? If so, why?
27. Another theme from the diaries was the use of GenAI was to make information more accessible or understandable. Do you use GenAI for this purpose? If so, why?
28. If you are comfortable doing so, can you go into more detail of whether your disability, neurodivergence, mental health condition, or physical health condition influences your use of GenAI?
29. In what ways does GenAI fall short of addressing your access needs?
30. “Are there any accessibility, mental health, physical health, or other needs/related tasks you would want to use GenAI but don’t yet for? If so, why don’t you use GenAI for them yet”?
31. What changes, if any, would you make to GenAI so that it would be more useful for meeting accessibility needs?
32. Do you do more or less of certain tasks since starting regular use of GenAI?
33. Are you satisfied overall with the use of GenAI in your life? Why or why not?
34. “Can you recall any specific moments when GenAI was particularly helpful? If yes: can you describe those moments”?
35. “Can you recall any specific moments when GenAI was particularly unhelpful? If yes: can you describe the moments? What did you do to overcome these challenges”?
36. How important is it to you that GenAI “get it right”?
37. If GenAI doesn’t get it right, do you continue to engage or move on?
38. What trade-offs do you make when using GenAI tools?
39. Were there any moments when you were emotionally engaged when using a GenAI tool?
40. How much have you been affected emotionally by GenAI outputs?
41. Was there any moment when you wanted to know what GenAI would generate or say next?
42. How many back-and-forths do your conversations with GenAI have?
43. Despite any challenges, what motivates you to continue using GenAI?
44. Were there any moments when you empathized with or connected with a GenAI tool?
45. How easy or difficult is using GenAI for you?
46. Did your use of GenAI ever impact how you interact with a friend, family member, coworker, stranger? If so, how?
47. Did anyone else ever notice your use of GenAI? If yes, did you feel comfortable talking about it with them?
48. How comfortable would you be disclosing what you use GenAI for with others?
49. Have there been moments when you’ve felt more comfortable sharing something with Generative AI than with other people?
50. Any other noteworthy social experiences you had related to your GenAI use?
51. In 5 years, how do you think GenAI will have changed, and what effects do you expect these changes to have on disabled people?
52. Those are all the questions I have for you. Is there anything about GenAI you would like to mention that we haven’t not covered so far but you’d like to tell us?
53. Anything else about your experience with using GenAI for accessibility you’d like to tell us?
54. Anything else you would like to share with me? Thanks! This is the end of the interview.
